# Systemic exposure to cisplatin and paclitaxel after intraperitoneal chemotherapy in ovarian cancer

**DOI:** 10.1007/s00280-023-04512-z

**Published:** 2023-03-09

**Authors:** Loek A. W. de Jong, Marie Lambert, Nielka P. van Erp, Lukas de Vries, Etienne Chatelut, Petronella B. Ottevanger

**Affiliations:** 1 Department of Pharmacy, Radboud University Medical Center Research Institute for Medical Innovation, Radboud University Medical Center, Geert Grooteplein Zuid 10, P.O. Box 9101, 6525 GA Nijmegen, The Netherlands; 2 Institut Claudius‑Regaud, and Université de Toulouse, Centre de Recherche en Cancérologie de Toulouse, Inserm, 1, avenue Irène Joliot‑Curie, Toulouse, France; 3 Department of Medical Oncology, Radboud University Medical Center Research Institute for Medical Innovation, Radboud University Medical Center, Nijmegen, The Netherlands

**Keywords:** Ovarian cancer, Cancer chemotherapy, Intraperitoneal, Pharmacokinetics, Cisplatin, Paclitaxel

## Abstract

**Purpose:**

To determine the systemic exposure to cisplatin and paclitaxel after adjuvant intraperitoneal administration in patients with advanced ovarian cancer who underwent primary debulking surgery. This could provide an explanation for the high incidence of systemic adverse events associated with this treatment regimen.

**Methods:**

This is a prospective pharmacokinetic study in patients with newly diagnosed advanced ovarian cancer who were treated with intraperitoneal administered cisplatin and paclitaxel. Plasma and peritoneal fluid samples were obtained during the first treatment cycle. The systemic exposure to cisplatin and paclitaxel was determined and compared to previously published exposure data after intravenous administration. An exploratory analysis was performed to investigate the relation between systemic exposure to cisplatin and the occurrence of adverse events.

**Results:**

Pharmacokinetics of ultrafiltered cisplatin were studied in eleven evaluable patients. The geometric mean [range] peak plasma concentration (C_max_) and area under the plasma-concentration time curve (AUC_0–24 h_) for cisplatin was 2.2 [1.8–2.7] mg/L and 10.1 [9.0–12.6] mg h/L, with a coefficient of variation (CV%) of 14 and 13.0%, respectively. The geometric mean [range] observed plasma concentration of paclitaxel was 0.06 [0.04–0.08] mg/L. No correlation was found between systemic exposure to ultrafiltered cisplatin and adverse events.

**Conclusion:**

Systemic exposure to ultrafiltered cisplatin after intraperitoneal administration is high. In addition to a local effect, this provides a pharmacological explanation for high incidence of adverse events seen after intraperitoneal administration of high-dose cisplatin. The study was registered at ClinicalTrials.gov under registration number NCT02861872.

**Supplementary Information:**

The online version contains supplementary material available at 10.1007/s00280-023-04512-z.

## Introduction

Ovarian cancer is a common type of cancer of the female reproductive system. Globally approximately 295,000 new diagnoses and 185,000 deaths were reported in the year 2018, which reflects a mortality-to-incidence ratio of approximately 0.6 which is the highest of all gynaecological cancers [1]. As a result of non-specific and/or absence of symptoms at early disease stage, ovarian cancer is often diagnosed at a late stage in which the cancer has spread beyond the ovary. The peritoneum is the most common site for metastases. Treatment for advanced-stage epithelial ovarian cancer, including fallopian tube and primary peritoneal cancers, involves primary debulking surgery followed by adjuvant platinum-based chemotherapy. When primary debulking surgery is not feasible, neoadjuvant chemotherapy followed by interval debulking surgery and adjuvant chemotherapy can be performed. For all patients a total of 6 cycles of platinum-taxane combination is recommended [2]. After completion of chemotherapy, maintenance therapy with poly adenosine diphosphate-ribose polymerase (PARP) inhibitors with or without bevacizumab improves long-term progression-free survival [3, 4].

Although first-line platinum-based chemotherapy is considered standard of care for advanced-stage epithelial ovarian cancer, a major controversy concerns the route of administration which can be intravenous or intraperitoneal. Intraperitoneal administration results in increased drug concentrations and exposure time at the target site, which is considered beneficial for the efficacy of these cytotoxic drugs. Despite the theoretical pharmacological advantage, the evidence for clinical benefit of intraperitoneal administration of chemotherapy after primary debulking surgery from phase III trials is inconclusive. Until now, four pivotal phase III trials have been performed investigating different agents, doses and schedules [5–8]. Three of these trials showed an increased median overall survival (OS) for intraperitoneal treatment over intravenous treatment with a median OS benefit ranging from 8 to 16 months [5–7]. More recently, a large phase III trial in 1560 patients showed no difference in median progression free survival between two intraperitoneally regimens (cisplatin or carboplatin) and dose-dense intravenous paclitaxel and carboplatin [8]. The incorporation of bevacizumab in all arms of this study, which was absent in the earlier trials, could have equalised or negated the effect of intraperitoneal chemotherapy administration. Also the dose of cisplatin and carboplatin was lower than in the older positive trials.

Negative consequences of intraperitoneal, mainly cisplatin, administration of chemotherapy are higher incidence of adverse events, catheter complications and higher costs compared to intravenous treatment. Adverse events associated with intraperitoneal administration of platinum-based chemotherapy includes both local and systemic events [5–7]. Local adverse events can be expected as a direct result of the intraperitoneal effects of the cytotoxic drugs and includes gastrointestinal events and abdominal pain. Systemic adverse events include bone marrow toxicity, renal toxicity, ototoxicity, neurological toxicity, fever, fatigue and metabolic events. Another important adverse event of intraperitoneal administration are catheter-related complications, including catheter infection, blocked catheter, leaking catheter and port access problems, which is the primary reason for discontinuation of therapy [9]. In clinical practice only 44% of patients who started intraperitoneal therapy are able to complete six or more cycles, compared to 91% of patients receiving intravenous therapy.

Intraperitoneal administration can be performed using different approaches. The first method is intraperitoneal administration using an indwelling catheter. The drug remains in the abdominal cavity after administration from where it will be absorbed. The second approach consists of perfusion of the drug immediately after cytoreductive surgery for several hours after which the chemotherapy is removed from the peritoneal cavity. This approach is often combined with hyperthermia, which is referred to as hyperthermic intraperitoneal chemotherapy (HIPEC). A recent developed intraperitoneal drug delivery technique is pressurized intraperitoneal aerosol chemotherapy (PIPAC) using a nebulizer. It can be expected that these different methods of intraperitoneal administration will impact the pharmacokinetics of the drugs. The majority of pharmacokinetic studies regarding intraperitoneal administration of chemotherapy in patients with gynaecological malignancies involve intraoperative administration. Although extensively studied in the past, the available full text articles in English regarding pharmacokinetics after intraperitoneal administration of cisplatin and paclitaxel using an indwelling catheter are considered sparse [10–13]. Although high systemic exposure of cisplatin can be expected after intraperitoneal administration, a proper comparison with pharmacokinetic data after intravenous administration is lacking thus far.

The current study investigated the systemic exposure to both cisplatin and paclitaxel after intraperitoneal administration in patients with ovarian cancer stage III receiving intraperitoneal chemotherapy after primary debulking surgery to provide an explanation for the high incidence of systemic adverse events associated with this treatment regimen. Knowledge about the systemic uptake of drugs administered intraperitoneally can help to find the most optimal intraperitoneal chemotherapy schedule that balance efficacy and safety of the treatment.

## Materials and methods

### Patients and data collection

Eligible patients aged between 18 and 70 years, with newly diagnosed stage III epithelial ovarian cancer, including fallopian tube and primary peritoneal cancer, with optimal or complete primary debulking (residual disease ≤ 1 cm), a World Health Organisation (WHO) performance status of 0–2, normal blood counts and adequate renal and hepatic function, who were planned to receive adjuvant intraperitoneal chemotherapy were enrolled.

Monitoring of haemoglobin, haematocrit, erythrocyte, thrombocyte and white blood cell (WBC) counts with differential classification of neutrophils, lymphocytes, monocytes, eosinophils and basophils, together with monitoring of liver panel, renal function and electrolytes took place as part of routine clinical care prior to the start and prior to day 8 of every treatment cycle. The occurrence of adverse events, other than laboratory abnormalities, that required a dose reduction or discontinuation of therapy were extracted from the medical record.

The study protocol was approved by the institutional ethics committee Arnhem-Nijmegen (Nijmegen) and was compliant with the Declaration of Helsinki. All patients provided written informed consent before entering the study. The study was conducted at the Radboud University Medical Center in Nijmegen, the Netherlands.

### Treatment plan

Patients were treated with adjuvant chemotherapy after primary debulking surgery according to GOG-172 [7] which is considered routine clinical care. The chemotherapy schedule consisted of 135 mg/m^2^ intravenous paclitaxel on day 1 in 16 h, 100 mg/m^2^ intraperitoneal cisplatin on day 2 and 60 mg/m^2^ intraperitoneal paclitaxel on day 8. This treatment schedule was administered every three weeks for a total of six cycles. Intraperitoneal administration was performed using a subcutaneous implantable port and catheter (Porth-a-Cath) system that was placed after debulking surgery. All intraperitoneally administered fluids were prewarmed to 37 °C before administration. Intraperitoneal cisplatin administration took around 1 h. The intraperitoneal administration schedule is provided in the supplementary file (Supplemental Table S2). Patients were treated with premedication and hyperhydration schedule according to local protocols.

### Pharmacokinetic sampling and analytical assay

Samples for assessment of pharmacokinetics were taken during the first cycle only, on day 2 and day 8. Blood samples were obtained from an indwelling canula in EDTA tubes as follows: before infusion, 15, 30 min, 1, 2, 4, 8, 16, 24 h after the end of the intraperitoneal chemotherapy infusion. A peritoneal fluid sample was taken through the Porth-a-Cath system at the end of intraperitoneal drug administration. Immediately after collection both blood and peritoneal fluid samples were centrifuged to separate plasma and debris in peritoneal fluid. An aliquot of plasma and peritoneal fluid was saved and stored at − 40 °C until analysis. For all cisplatin samples, plasma and peritoneal fluid was immediately ultrafiltered through a Centrifree^®^ ultrafiltration device with Ultracel^®^ PL membrane to separation unbound platinum in plasma and peritoneal fluid. The ultrafiltered cisplatin samples were stored at − 40 °C until analysis. Ultrafiltered and (total) plasma cisplatin concentrations were determined by a validated method using flameless atomic absorption spectroscopy [14]. Paclitaxel plasma and IP fluid concentrations were determined using a validated HPLC–MS/MS method [15].

### Population pharmacokinetic model

Cisplatin data (i.e., ultrafiltered peritoneal and plasma concentrations, and plasma protein bound concentrations obtained by difference between total and ultrafiltered plasma concentrations) were analyzed simultaneously by non-linear mixed effects modeling with NONMEM version 7.4.1 (ICON Development Solutions, Ellicott City, MD, USA) using Pirana^®^ software (version 2.9.9) as an interface. The population pharmacokinetic approach was applied to analyze simultaneously the cisplatin concentrations in peritoneal fluid (IP), plasma ultrafiltrate (UF), and the protein bound plasma (PB). The PB concentrations were obtained by deduction of the UF concentration measured to the total plasma measured concentrations. The analyses were performed using the First-Order Conditional Estimation with the Interaction (FOCE-I) option. The model development was based on objective function value (OFV = − 2 log likelihood), goodness-of-fit plots, relative standard error (RSE), estimate PK parameters and estimate residual error. For all analyses, interindividual variability (IIV) were modeled using an exponential model and proportional model was used to estimate residual variability.

Area-under-the-curve of ultrafiltered cisplatin plasma concentrations versus time from 0 to 24 h (AUC_0–24 h_) was calculated for each patient with NONMEM by integrating the amounts of the drug in a dummy compartment (using the ADVAN6 subroutine) ${\int }_{0}^{t}Ct \times dt$.

## Results

### Patients

Eleven patients were enrolled in the study between January 2018 and October 2019. Patient characteristics are summarised in Table 1.

**Table 1:** Patients’ characteristics

Characteristic	Median (range)
Age (years)	50 (35–60)
Weight (kg)	67 (57–116)
Height (cm)	169 (157–188)
Body mass index (kg/m^2^)	24.7 (20.2–39.7)
Body surface area (m^2^) ^a^	1.76 (1.63–2.35)
FIGO stage	
IIIb	2
IIIc	9
WHO performance status	
0	10
1	1
Gross residual disease	
No (≤ 1 cm)	11
Yes (> 1 cm)	0
Histologic type	
Serous adenocarcinoma	9
Endometrioid adenocarcinoma	1
Carcinosarcoma	1
Ascites present at primary debulking surgery	
≥ 1000 mL	1
< 1000 mL	1
None (≤ 100 mL)	2
Unknown	7
Interval between primary debulking surgery and first cycle (days)	34 (24–40)

^a ^Calculated using the Mosteller Formula

### Pharmacokinetics of ultrafiltered cisplatin

Plasma ultrafiltered and total cisplatin PK data were available from eleven patients. Intraperitoneal fluid samples were available from six patients at the end of cisplatin administration. A total of 147 samples (6 IP, 71 UF and 70 PB cisplatin concentrations) were used to estimate pharmacokinetics parameters. The final structural pharmacokinetic model included four compartment: three compartments with available concentrations (V_IP_, V_UF_, V_B_/f_bp_) and one peripheral compartment of distribution of UF cisplatin (Supplemental Fig S1) with f_bp_ being the fraction of plasma UF cisplatin mainly eliminated by protein binding. Due to identifiability issue, it was not possible to consider two elimination processes for plasma UF cisplatin. Interindividual variability had to be fixed to 0 for V_IP_, V_UF_, and K_30_. The population pharmacokinetic parameters of the final model are summarized in Supplementary Table 1. The Individual predicted and individual observed cisplatin concentrations for both ultrafiltered plasma and total plasma are shown in supplemental Fig S2 and S3, respectively.

**Fig. 1:**
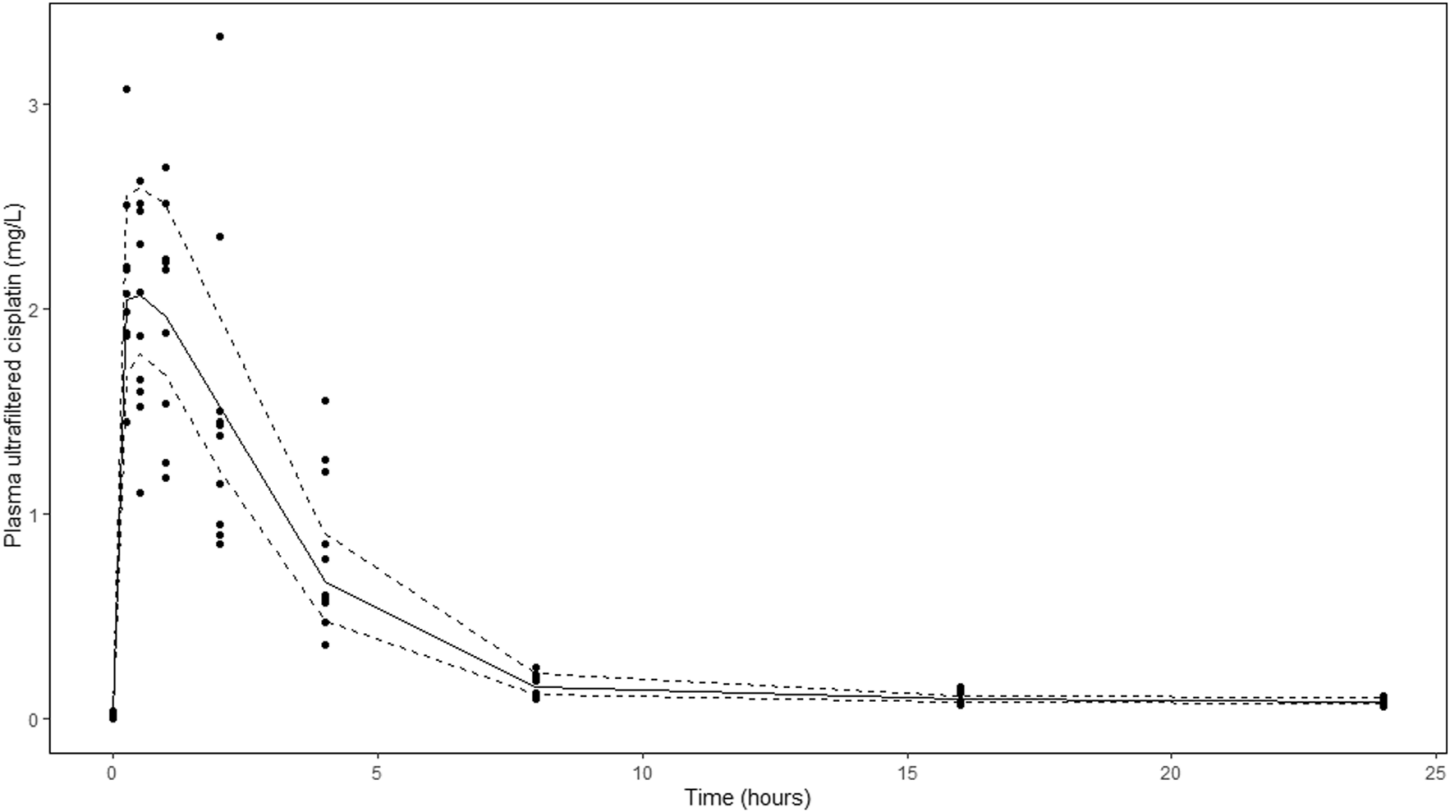
PK POP plasma ultrafiltered cisplatin concentration vs. time curve including individual observations (∙) and 5th, 50th, 95th percentiles of plasma ultrafiltered cisplatin individual prediction

The PopPK plasma ultrafiltered cisplatin concentration vs. time curve including individual observations is shown in Fig. 1. The geometric mean [range] ultrafiltered cisplatin plasma C_max_, T_max_ and area under the curve (AUC_0–24 h_) was 2.2 [1.8–2.7] µg/mL, 1.9 [1.5–2.9] hours and 10.1 [9.0–12.6] µg h/mL, respectively. Interpatient variability for C_max_, T_max_ and AUC _0–24 h_ was characterised by a coefficient of variation (CV%) for ultrafiltered cisplatin of 13.7, 20.2 and 13.0%, respectively. The mean (± sd) ratio of the C_max_ in peritoneal fluid to the C_max_ in plasma was 29 ± 4.

### Pharmacokinetics of paclitaxel

PK data were available from six patients. An intraperitoneal fluid sample at the end of paclitaxel administration was available from only one patient. Due to limited paclitaxel samples it was not possible to create a population pharmacokinetic model for paclitaxel. The minimal available data for paclitaxel were compared with earlier published data. Blood sampling took place between 1.5 and 3.1 h after administration. For paclitaxel geometric mean [range] observed plasma concentration was 0.06 [0.04–0.08] µg/mL. Due to limited short-term sampling in combination with paclitaxel’s ability to maintain high drug levels in the peritoneal cavity for several days, it was not possible to calculate a reliable AUC for paclitaxel in the plasma compartment. The ratio of the concentration observed in the single collected peritoneal fluid sample to the geometric mean concentration in plasma was 972.

The pharmacokinetic data for the individual patients for cisplatin and paclitaxel is summarised in Table 2.

**Table 2:** Pharmacokinetics after intraperitoneal administration of cisplatin and paclitaxel in plasma and peritoneal fluid

Patient #	Cisplatin ^a^										Paclitaxel ^b^			
	Dose (mg)	Infusion time (min)	Ultrafiltered plasma			Total plasma			Ultrafiltered peritoneal fluid		Dose (mg)	Plasma		Peritoneal fluid
			C_max_ (µg/mL)	T_max_ (h)	AUC_0–24 h_ (µg h/mL)	C_max_ (µg/mL)	T_max_ (h)	AUC_0–24 h_ (µg h/mL)	C_max_ (µg/mL)	T_max_ (h)		Conc (µg/mL)	T (h)	Conc (µg/mL)
Patient 1	180	57	2.0	1.6	9.2	4.9	5.0	99.3	62.6	0.8	110	0.06	2.5	n/a
Patient 2	200	73	2.0	2.9	10.9	3.8	4.6	74.0	65.9	1.1	120	n/a	n/a	58.3
Patient 3	160	47	2.1	1.7	9.0	3.6	3.3	69.1	56.6	0.6	100	n/a	n/a	n/a
Patient 4	170	50	1.8	2.1	9.9	3.9	5.1	78.7	61.7	0.8	105	0.04	5.2	n/a
Patient 5	170	43	2.3	1.5	9.0	3.9	2.1	78.5	55.8	0.7	100	n/a	n/a	n/a
Patient 6	210	58	2.4	1.8	11.9	4.6	5.2	90.2	73.7	0.8	125	0.06	1.5	n/a
Patient 7	170	65	1.8	1.9	9.0	4.2	5.3	83.3	55.6	1.1	100	n/a	n/a	n/a
Patient 8	240	53	2.4	1.7	9.5	4.0	3.2	77.8	77.2	0.9	145	n/a	n/a	n/a
Patient 9	170	70	2.3	2.2	11.6	5.4	5.4	107.1	51.8	1.2	100	0.06	2.8	n/a
Patient 10	230	47	2.7	1.7	12.6	4.8	3.5	93.5	83.7	0.6	140	0.08	3.1	n/a
Patient 11	160	50	1.9	2.1	9.3	3.6	5.1	72.1	54.5	0.8	100	0.07	5.2	n/a
Geometric mean [range]	185 [160–240]	55 [43–73]	2.2 [1.8–2.7]	1.9 [1.5–2.9]	10.1 [9.0–12.6]	4.3 [3.6–5.4]	4.3 [2.1–5.4]	84.0 [69.1–107.1]	62.8 [51.8–83.7]	0.9 [0.6–1.2]	112 [100–145]	0.06 [0.04–0.08]	3.1 [1.5–5.2]	n/a

^a^ For cisplatin, the individual predicted PK parameters are shown. ^b^ For paclitaxel, the observed concentrations are provided

*n/a* not available, due to missing or limited samples pharmacokinetic parameter could not be evaluated

### Tolerability and toxicity

Three out of eleven patients (27%) completed the total of six chemotherapy courses, of whom two patients required a dose reduction. Three patients completed a total of five cycles, three patients completed three cycles, and two patients completed only one cycle of intraperitoneal chemotherapy. Reasons for not completing the full planned intraperitoneal chemotherapy were the occurrence of decline in renal function, neutropenia, nausea and vomiting, cardiac problems and bacterial peritonitis. One patient was treated with three cycles of neo-adjuvant intravenous carboplatin and paclitaxel prior to intraperitoneal administration and therefore received only three intraperitoneal chemotherapy cycles. The occurrence of haematologic and renal toxicity after intraperitoneal chemotherapy is presented in Table 3. Approximately half of the patients (55%) experienced grade 3/4 neutropenia and 18% experienced grade 3/4 WBC toxicity. Grade 3/4 renal toxicity, anaemia or thrombocytopenia did not occur in any of the patients. In an exploratory analysis no correlations were found between systemic exposure to ultrafiltered cisplatin and decreased neutrophil count and/or increased creatinine levels (data not shown).

**Table 3:** Haematologic and renal toxicity after intraperitoneal chemotherapy

Adverse drug event ^a^	Number of patients (% of total)
Anemia	
Any	11 (100)
Severe (grade 3 and 4)	0 (0)
Creatinine increased	
Any	3 (27)
Severe (grade 3 and 4)	0 (0)
Neutrophil count decreased	
Any	10 (91)
Severe (grade 3 and 4)	6 (55)
Platelet count decreased	
Any	7 (64)
Severe (grade 3 and 4)	0 (0)
White blood cell decreased	
Any	10 (91)
Severe (grades 3 and 4)	2 (18)

^a ^Graded according to CTCAE v5.0 [16]

## Discussion

This study demonstrates that intraperitoneal administration of 100 mg/m^2^ cisplatin results in high systemic exposure to ultrafiltered cisplatin. It is demonstrated that systemic uptake from the peritoneal cavity is highly drug dependent. This study provides an explanation for the high incidence of systemic adverse events associated with this high-dose cisplatin-based intraperitoneal therapy. However, in addition to a local treatment effect, the systemic ultrafiltered cisplatin exposure might also contribute to the survival benefit seen with intraperitoneal cisplatin-based chemotherapy in ovarian cancer.

Systemic exposure to unbound cisplatin is closely correlated with tumour response [17]. The AUC_0–24 h_ of 10.1 µg h/mL for ultrafiltered cisplatin after intraperitoneal administration of 100 mg/m^2^ cisplatin measured in our study is considered high compared to the AUC_0–24 h_ after an equal intravenous dose, which is 5.8–8.4 µg h/mL [18–21]. Although it is challenging and questionable to compare these results, the patient population in these studies is considered comparable with the patients in our study and mainly consist of female patients with advanced gynaecological cancers. Our findings are in line with an earlier study that found an AUC_0–24 h_ to ultrafiltered cisplatin after intraperitoneal administration of 90 mg/m^2^ of 8.1 µg h/mL [10]. The C_max_ of ultrafiltered cisplatin after an intravenous cisplatin dose of 100 mg/m^2^ is highly dependent on the infusion time. Previously published data demonstrated C_max_ values after a bolus infusion (5–8 min), a 30-min infusion, a 3-h infusion and a 24-h infusion of 14.2, 6.0, 2.9 and 0.4 µg/mL, respectively [18, 21]. Taking into account the peritoneal administration time of cisplatin of 55 [43–73] min, the observed C_max_ of ultrafiltered cisplatin in plasma of 2.15 µg/mL after intraperitoneal administration seems lower compared to intravenously administered cisplatin data. Although a small water-soluble molecule like cisplatin is expected to be absorbed rapidly, the lower C_max_ in plasma can be explained as a result of gradual uptake from the peritoneal compartment in the blood stream. This is also reflected by the T_max_ that ranges between 1.5 and 2.9 h after the start of intraperitoneal infusion. The interpatient variability for both C_max_ and AUC_0–24 h_ after intraperitoneal administration of cisplatin was much lower compared to earlier findings reporting a CV% of 58% and 63% for C_max_ and AUC_0–24 h_, respectively [10]. It was even lower compared to interpatient variability after intravenous administration describing a CV% of 31% and 33–34% for C_max_ and AUC_0–24 h_, respectively [18–21]. Although intraperitoneal administration is more complex compared to intravenous administration, the pharmacokinetic variability seems low. The total cisplatin C_max_ and T_max_ in plasma exceeds the C_max_ and T_max_ of ultrafiltered cisplatin. As seen in our study, the absorption from the peritoneal compartment towards the bloodstream is expected to continue until 4–5 h after intraperitoneal administration. Due to high reactivity of ultrafiltered cisplatin, the T_max_ for ultrafiltered cisplatin in plasma is reached shortly after intraperitoneal administration. Thereafter the absorption from the peritoneal compartment is outweighed by binding and excretion of ultrafiltered cisplatin in blood plasma.

The high systemic exposure to ultrafiltered cisplatin after intraperitoneal administration might be caused by higher fraction of complexed cisplatin to low weight molecules in the ultrafiltered part. When absorbed from the peritoneal cavity to the systemic compartment, cisplatin crosses epithelial barriers. A fraction of diaquaplatin, the active intermediate formed from cisplatin, is likely to be complexed to low-molecular weight molecules such as glutathione, cysteine and methionine before it reaches the blood stream. This complexed cisplatin is considered as inactive drug, but still contribute to the total amount of ultrafiltered cisplatin measured in plasma. Therefore the AUC_0–24 h_ is expected to be an overestimation of active cisplatin.

Taking into account the high systemic exposure to ultrafiltered cisplatin after intraperitoneal administration, it is remarkable that the phase III studies supporting the use of intraperitoneal cisplatin uses equal [5] or even higher intraperitoneal doses [6, 7] in the intervention group compared to the control group. All of the clinical studies showing an increased median OS for intraperitoneal cisplatin over intravenous cisplatin investigated a dose of 100 mg/m^2^ [5–7]. The clinical trial that failed to show a survival advantage for intraperitoneal cisplatin investigated a lower dose of 75 mg/m^2^ [8]. The local effect of cisplatin is expected to be both exposure-time and concentration dependent. However, the current intraperitoneal concentrations of 52–84 µg/mL reached after intraperitoneal administration of 100 mg/m^2^ cisplatin, highly exceeds the IC_50_ values for cisplatin in ovarian cancer organoids [22]. This suggests that a dose reduction of cisplatin would not necessarily deteriorate its local antitumor effect. In addition to a local effect, the systemic exposure might be driving the clinical benefit seen with 100 mg/m^2^ cisplatin-based intraperitoneal chemotherapy.

High systemic exposure to ultrafiltered cisplatin is expected to cause increased incidence of systemic adverse events like peripheral neuropathy, haematological toxicity and ototoxicity [23, 24]. The landmark phase III iv/ip studies differ in terms of adverse events [5–8]. In the clinical trial that forms the basis of the intraperitoneal scheme used in this study, significantly more patients in the intraperitoneal therapy group than in the intravenous therapy group suffered from grade 3/4 WBC toxicity (76% vs. 64%), thrombopenia (12% vs. 4%), neurologic events (19% vs. 9%), fatigue (18% vs. 4%) and metabolic events (27% vs. 7%) [7]. The findings of this study suggest that this might be the result of high systemic exposure to ultrafiltered cisplatin. Lowering the dose of cisplatin to 75 mg/m^2^ is found to decrease the incidence of systemic adverse events [8].

In contrast to cisplatin, a large water insoluble drug like paclitaxel is very slowly absorbed from the peritoneal compartment. It has been demonstrated that the pharmacological advantage, very slow peritoneal clearance and a high peritoneal-plasma AUC ratio of 996 [12], can be attributed to the presence of the solvent vehicle Cremophor EL [25]. Paclitaxel has a specific mode of action in which cytotoxicity is related to the duration of exposure [26]. Given its long intraperitoneal residence time paclitaxel might also be cytotoxic to tumour cells that undergo replication at later time points after initial administration which might improve efficacy over intravenous administration of paclitaxel. Systemic uptake is around 30% after intraperitoneal administration, resulting in a much lower systemic exposure [25]. The C_max_ in plasma found in our study after intraperitoneal administration of 60 mg/m^2^ paclitaxel was 0.06 µg/mL and is in line with earlier findings of 0.04–0.69 µg/mL for C_max_ [12, 13, 25]. The major adverse events of paclitaxel are neurotoxicity and bone marrow toxicity, in particular neutropenia. Both are thought to be related to the systemic exposure of paclitaxel [27]. An intraperitoneal paclitaxel dose of 60 mg/m^2^ is not expected to cause severe systemic adverse events. Nevertheless, local adverse events can be expected, given the long local residence time after intraperitoneal administration. Since intraperitoneal administration of paclitaxel is investigated as an addition to systemic therapy instead of replacing it, it can still contribute to a higher rate of grade 3 and 4 systemic toxicities as is seen in clinical studies [7]. In general we speculate that systemic adverse events after intraperitoneal administration of cisplatin and paclitaxel is a result of high systemic exposure to ultrafiltered cisplatin and that local adverse events may be due to intraperitoneal paclitaxel.

Soundness of this study are its prospective nature, rich cisplatin blood sampling and the accurate time information for the blood samples. This is essential to accurately determine the pharmacokinetic parameters. The comparison with systemic exposure data after intravenous administration is helpful to place the findings of the study in perspective. Although this study yielded new insights in intraperitoneal drug delivery, there are some shortcomings that need to be addressed. First, since patients were allowed to leave the hospital after administration of paclitaxel, few paclitaxel samples could be collected beyond 12 h. Since paclitaxel is very slowly absorbed from the peritoneal compartment sampling at later timepoints is recommended for an accurate estimation of the systemic exposure. Second, since only one intraperitoneal fluid sample was collected, it was not possible to determine total exposure in the peritoneal compartment. Also peritoneal sampling through the PAC turned out to be more challenging than expected. The tube was often blocked while drawing up intraperitoneal fluid making it impossible to collect an intraperitoneal sample for the majority of patients. However the high C_max_ peritoneal to plasma ratio, which is especially the case for paclitaxel, demonstrates the pharmacological advantage of intraperitoneal administration resulting in high local drug concentrations. Third, there turned out to be a delay in sampling time causing the first ultrafiltered cisplatin samples to be taken after the C_max_. However, using the population pharmacokinetic model we were still able to predict the maximum plasma concentration for each individual patient.

The results of this study together with earlier findings in literature shed a new light on intraperitoneal chemotherapy administration. The importance of systemic exposure of intraperitoneal administrated drugs is often underestimated. We showed that the assumption that systemic exposure after intraperitoneal therapy remains low does not apply for all cytotoxic drugs and in some circumstances can be very high, as is the case for intraperitoneal administered cisplatin. The chemotherapy schedule used in this study is the GOG-172 regimen that demonstrated a 16-month improvement in median overall survival [7]. However, in clinical practice it has been demonstrated that a large proportion of patients receive a modified regimen at treatment initiation, including cisplatin dose reductions, replacement of cisplatin with carboplatin and/or substitution of intravenous paclitaxel [28]. Together with the high incidence of grade 3/4 AEs this demonstrates that there is still room for improvement. This study clearly showed that, in addition to a local effect, both the clinical benefit and systemic adverse events seen with cisplatin-based intraperitoneal chemotherapy might be a result of high systemic exposure to ultrafiltered cisplatin.

Intraperitoneal administration of 100 mg/m^2^ cisplatin results in high systemic exposure to ultrafiltered cisplatin. Interindividual pharmacokinetic variation of ultrafiltered cisplatin after intraperitoneal administration is remarkably low. Systemic uptake from the peritoneal cavity is highly drug dependent, with cisplatin showing fast peritoneal-to-blood transport, whereas paclitaxel is very slowly absorbed. The long intraperitoneal residence time of paclitaxel might be related to the local toxicity of intraperitoneal administration.

## Supplementary Information

Below is the link to the electronic supplementary material.

Supplementary file1 (DOCX 27 KB)

Supplementary file2 Fig S1. The final structural pharmacokinetic model. Ka = absorption constant, VIP = peritoneal volume, VUF = ultrafiltered plasma volume, K24 = transfer constant between ultrafiltered plasma compartment and peripheral compartment, K42 = transfer constant between peripheral compartment and ultrafiltered plasma compartment, K23 = plasma protein binding constant expressed, K30 = elimination constant of cisplatin bound, VB/fbp= apparent bound plasma volume (TIF 54 KB)

Supplementary file3 Fig S2. Individual predicted and individual measured cisplatin concentrations for ultrafiltered plasma. The dashed lines represent the individual predicted concentrations and the solid lines represent the observed cisplatin concentrations. (TIF 48 KB)

Supplementary file4 Fig S3. Individual predicted and individual measured cisplatin concentrations for total plasma. The dashed lines represent the individual predicted concentrations and the solid lines represent the observed cisplatin concentrations. (TIF 46 KB)

## Data Availability

The data that support the findings of this study are available on request from the corresponding author. The data are not publicly available due to their containing information that could compromise the privacy of research participants.
