# Supplementary material for: Systemic exposure to cisplatin and paclitaxel after intraperitoneal chemotherapy in ovarian cancer
Source: Cancer Chemother Pharmacol. 2023 Mar 9;91(3):247–56. doi: 10.1007/s00280-023-04512-z (PMC10033566; doi:10.1007/s00280-023-04512-z)
Supplement: Supplementary file 1 — Supplementary file1 (DOCX 27 KB) [file 280_2023_4512_MOESM1_ESM.docx]

**Supplementary information**

**Systemic exposure to cisplatin and paclitaxel after intraperitoneal chemotherapy in ovarian cancer**

Loek AW de Jong^1^ *PharmD **, Marie Lambert^2^ *PharmD **, Nielka P van Erp^1^ *PharmD, PhD*, Lukas de Vries^1^, Etienne Chatelut^2^ *PharmD, PhD*, Petronella B Ottevanger^3^ *MD,* *PhD*.

^1^ Radboudumc, Department of Pharmacy, Radboud Institute for Health Sciences, P.O. Box 9101, 6500 HB Nijmegen, the Netherlands

**^2^** Institut Claudius‑Regaud, and Université de Toulouse, Centre de Recherche en Cancérologie de Toulouse, Inserm, 1, avenue Irène Joliot‑Curie, Toulouse, France

^3^ Radboudumc, Department of Medical Oncology, Radboud Institute for Health Sciences, P.O. Box 9101, 6500 HB Nijmegen, the Netherlands

* LJ and ML should be considered joint first author.

Corresponding author: Loek AW de Jong, Geert Grooteplein Zuid 10, 6525GA, Nijmegen, The Netherlands. Tel. +31 24 361 6405; Fax. +31 24 366 8755; E-mail: Loek.deJong@radboudumc.nl

**Supplemental Table S1. Population pharmacokinetic parameter corresponding to the final model.**

| **Parameters** | **Units** | **Final model (% RSE) [shrinkage]** |
| --- | --- | --- |
| Ka | h^-1^ | 0.64 (14.2) |
| V_IP_ | L | 2.26 (7.3) |
| V_UF_ | L | 27.2 (17.1) |
| V_B_/f_bp_ | L | 20.3 (14.2) |
| K24 | h^-1^ | 0.406 (29.6) |
| K42 | h^-1^ | 0.0263 (41.4) |
| K23 | h^-1^ | 0.394 (9.4) |
| K30 | h^-1^ | 0.033 (17.7) |
|  |  |  |
| IIV Ka | % | 24.5 (31.6) [12.2] |
| IIV K24 | % | 35.6 (28.5) [13.4] |
| IIV K42 | % | 9.1 (68.4) [67.8] |
| IIV K23 | % | 6.4 (63.4) [60.4] |
| IIV V_B_/f_bp_ | % | 18.2 (33.1) [17.6] |
|  |  |  |
| ε peritoneal | % | 14.2 (64.4) [19.2] |
| ε UF plasma | % | 29.2 (31.2) [7.4] |
| ε bound plasma | % | 26.1 (23.5) [6.9] |

K_a_ = absorption constant, V_IP_ = peritoneal volume, V_UF_ = ultrafiltered plasma volume, K24 = transfer constant between ultrafiltered plasma compartment and peripheral compartment, K42 = transfer constant between peripheral compartment and ultrafiltered plasma compartment, K23 = plasma protein binding constant expressed, K30 = elimination constant of cisplatin bound, V_B_/f_bp_= apparent bound plasma volume, IIV = interindividual variability, ε = proportional residual error

**Supplemental Table S2. Intraperitoneal treatment schedule.**

| **Drug** | **Volume (mL)** | **Administration time (min)^1^** |
| --- | --- | --- |
| *Intraperitoneal Cisplatin (day 2 of each cycle)* | | |
| Sodium Chloride 0.9% | 500 | 15-20 |
| Cisplatin 100mg/m^2^ in Sodium Chloride 0.9% | 1000 | 45-70 |
| Sodium Chloride 0.9% | 500 | 15-20 |
| *Intraperitoneal Paclitaxel (day 8 of each cycle)* | | |
| Sodium Chloride 0.9% | 1000 | 45 |
| Sodium Chloride 0.9% | 250 | 10-15 |
| Paclitaxel 60mg/m2 in Sodium Chloride 0.9% | 250 | 45 |
| Sodium Chloride 0.9% | 500 | 10-15 |

^1^ Administration was performed as rapidly as possible but administration time differs between patients.
