# Supplementary figures and images for: Systemic exposure to cisplatin and paclitaxel after intraperitoneal chemotherapy in ovarian cancer
Source: Cancer Chemother Pharmacol. 2023 Mar 9;91(3):247–56. doi: 10.1007/s00280-023-04512-z (PMC10033566; doi:10.1007/s00280-023-04512-z)

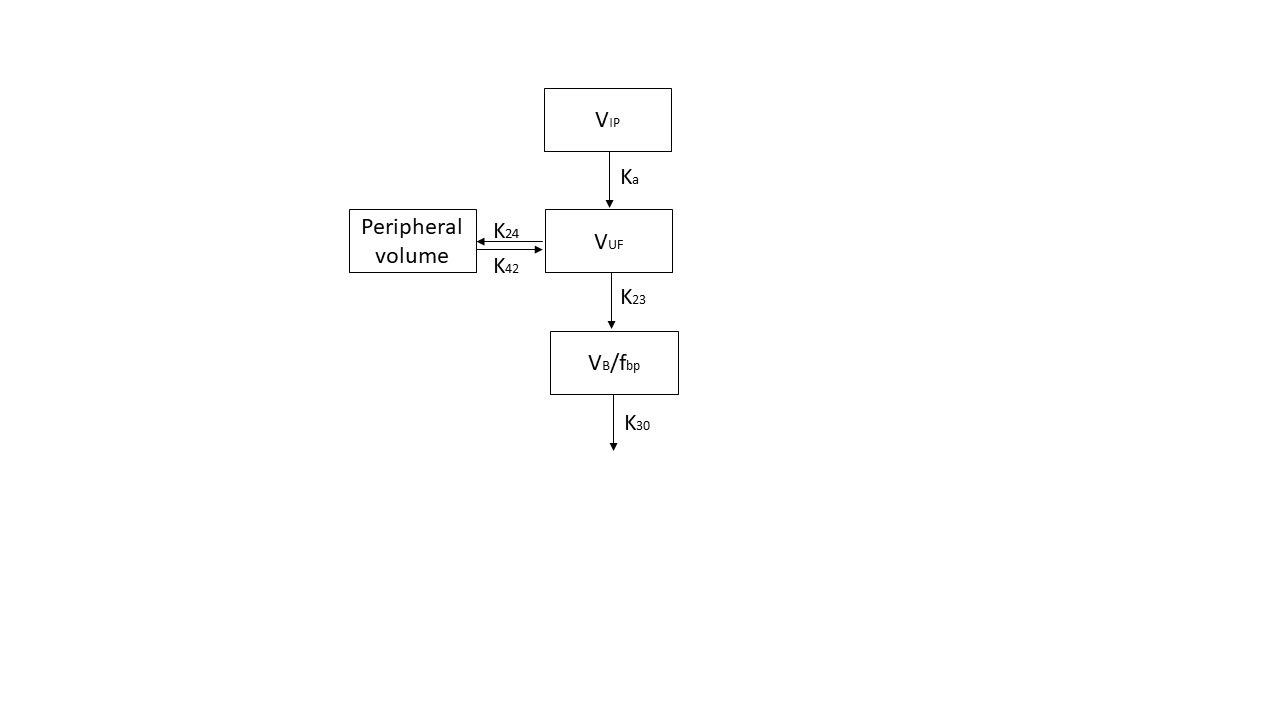

Supplement: Supplementary file 2 — Supplementary file2 Fig S1. The final structural pharmacokinetic model. Ka = absorption constant, VIP = peritoneal volume, VUF = ultrafiltered plasma volume, K24 = transfer constant between ultrafiltered plasma compartment and peripheral compartment, K42 = transfer constant between peripheral compartment and ultrafiltered plasma compartment, K23 = plasma protein binding constant expressed, K30 = elimination constant of cisplatin bound, VB/fbp= apparent bound plasma volume (TIF 54 KB) [file 280_2023_4512_MOESM2_ESM.tif]

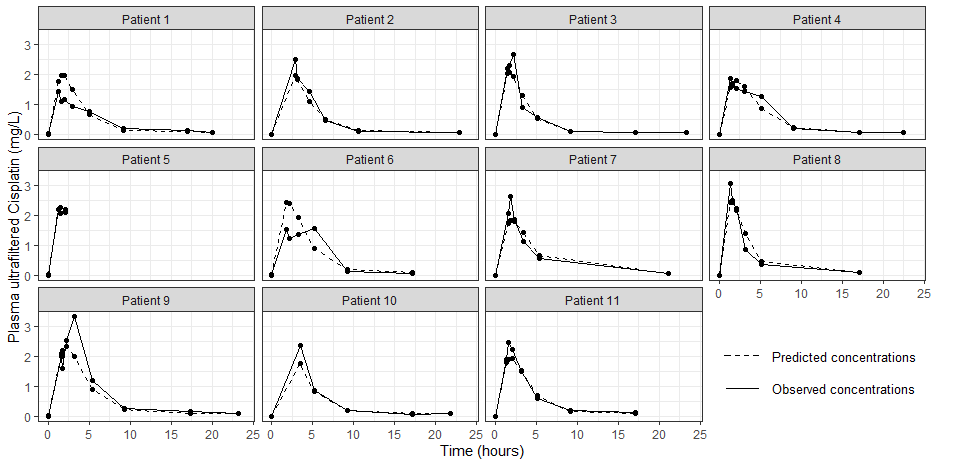

Supplement: Supplementary file 3 — Supplementary file3 Fig S2. Individual predicted and individual measured cisplatin concentrations for ultrafiltered plasma. The dashed lines represent the individual predicted concentrations and the solid lines represent the observed cisplatin concentrations. (TIF 48 KB) [file 280_2023_4512_MOESM3_ESM.tif]

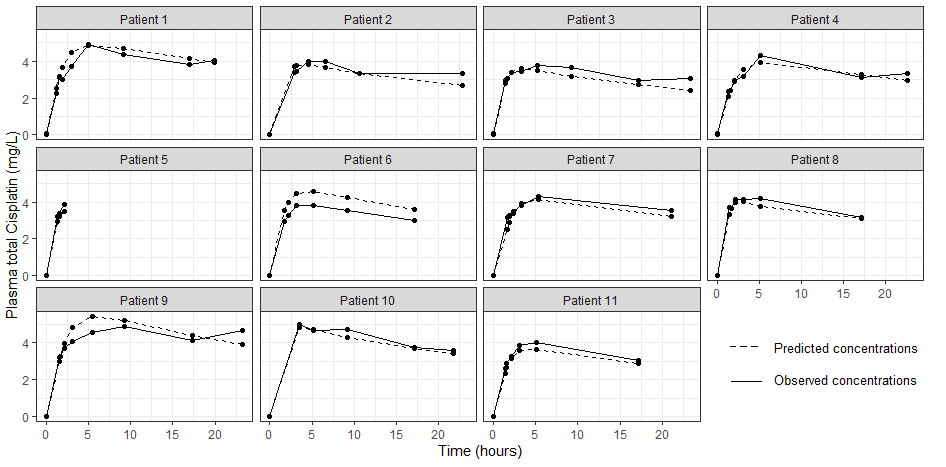

Supplement: Supplementary file 4 — Supplementary file4 Fig S3. Individual predicted and individual measured cisplatin concentrations for total plasma. The dashed lines represent the individual predicted concentrations and the solid lines represent the observed cisplatin concentrations. (TIF 46 KB) [file 280_2023_4512_MOESM4_ESM.tif]
